# Thermally Sprayed Coatings for the Protection of Industrial Fan Blades

**DOI:** 10.3390/ma17163903

**Published:** 2024-08-07

**Authors:** Maria Richert

**Affiliations:** Management Faculty, AGH University in Krakow, 30-067 Kraków, Poland; mrichert@agh.edu.pl

**Keywords:** thermal spraying, coatings, tungstem carbides, chromium carbides, wear erosion

## Abstract

This paper presents a study on thermally sprayed coatings. Coatings produced by high-velocity oxygen–fuel spraying HVOF and plasma spraying deposited on the A03590 aluminum casting alloy are tested. The subject of this research concerns coatings based on tungsten carbide WC, chromium carbide Cr_3_C_2_, composite coatings NiCrSiB + 2.5%Fe + 2.5%Cr, mixtures of tungsten and chromium powders WC-CrC-Ni, mixtures of carbide powders with the Cr_3_C_2_-NiCr + the composite 5% NiCrBSi and WC-Co + 5% NiCrBSi. The aim of this research is to find a coating most resistant to the erosive impact of particles contained in the medium centrifuged by industrial rotors. The suitability of the coating is determined by its high level of microhardness. The hardest coatings are selected from the coatings tested and subjected to abrasion tests against a sand particle impact jet and the centrifugation of a medium with corundum particles. It is found that the most favorable anti-erosion properties are demonstrated by a coating composed of a mixture of tungsten carbide and chromium carbide WC-CrC-Ni powders. It is concluded that the greatest resistance of this coating to the erosive impact of the particle jet results from the synergistic enhancement of the most favorable features of both cermets.

## 1. Introduction

Anti-erosion and anti-corrosion coatings are commonly used to protect metallic surfaces [1]. They provide protection against the destructive effects of phenomena such as environmental influence, abrasion, particle impact, friction and corrosion. These phenomena often occur simultaneously. Various types of coatings are used to protect surfaces exposed to damage. Numerous friction pairs in machines, tools, devices or machine parts are covered with special anti-corrosion coatings or layers. The hardening of the surface itself increases the resistance to wear and friction, an example of which is steel nitriding [2]. Surface protection methods are individually selected for particular tools, devices and other friction pairs.

Anti-erosion coatings can be produced using various methods. One of them is pulsed magnetron sputtering [3]. Other methods include the vacuum cathodic arc deposition system (FCVA) [4], physical vacuum deposition (PVD) [5] and chemical deposition in vacuum (CVD) [6]. Various thermal spray methods are also used [7,8,9,10]. The latest development in surface engineering is high-entropic materials used as anti-erosion coatings [11]. Thermal spray methods are often used for anti-erosion and anti-corrosion surface protection due to their low costs and multiple variations in spray techniques. Thermal spray methods and their applications have been presented in many publications on surface protection [12,13,14]. There are several methods of applying thermal spray coatings, such as oxy fuel wire (OFW), electric arc wire (EAW), oxy fuel powder (OFP), plasma arc powder (PAP) and high-velocity oxy fuel (HVOF) [15,16,17,18].

Thermal spray coatings are a widespread surface engineering technique used all over the world. The coating can be deposited in a molten or plastic state through propulsion of particles or through the impact onto a workpiece. The particles of powders sprayed in thermal spray methods, e.g., CoCrNiWC and CoCr-based intermetallics, have a melting point of approximately 1230–1600 °C. In turn, particles of NiCrSiB have a melting point of approximately 1050 °C. The CrC–NiCr and CrC–Ni-based particles melt at a temperature of approximately 1930 °C, and WC–Co, WC–Ni and WC-based particles melt at approximately 1250–1480 °C. Ceramic particles melt at temperatures above 2000 °C. Thermal spray technologies must provide a sufficiently high temperature to melt the particles.

Plasma spraying uses a plasma-forming gas, which is usually argon with possible additions of other elements. The plasma-forming gas is ionized under the electric arc and turns into plasma. The plasma escapes through a hole in the torch front along with the heated metallic or ceramic powder. The plasma arc has a temperature in the range of 1173–3730 °C (4000 ÷ 12,000 K), but it can reach up to 14,730 °C (15,000 K), depending on the distance from the burner nozzle [19,20].

In the gas jet, individual particles achieve high speeds: approx. 160 m/s in flame spraying and arc spraying, approx. 200–250 m/s in plasma spraying, and over 330 m/s in supersonic spraying. The high temperature causes melting of the particles, but as they reach the surface, they often cool below the melting point, which is the reason for the presence of unmelted particles in the coating structure. The low heat capacity of the particles falling on the substrate causes them to cool very quickly. It was found that the sprayed object did not heat up to a temperature higher than 100 °C. As a result of this characteristic process, a specific structure of the sprayed coating is created. Layers with a strongly differentiated structure are formed, with grains flattened along the sprayed surface. The layer structure may contain voids, porosity, delamination, spalling, interface contamination, transverse cracks, inter-lamellar pores, intra-lamellar cracks, unmelted particles, pull-outs, oxides, oxide clusters, oxide stringers or metallic inclusions. A high density of artifacts in the layer structure weakens its properties, such as hardness or abrasion resistance.

A major challenge in surface engineering is protecting the surfaces of industrial fan blades. The blades are exposed to intense impacts from particles, dust and atmospheric corrosion. Carbide coatings, such as WC, WC-Co(Cr) and Cr-Cr_3_C_2_, are used for anti-erosion protection of the blade surfaces. These types of coatings are also used in friction pairs and as anti-wear coatings in elements exposed to particle impact [21]. In turn, TiN and TiCrN [22,23,24,25,26,27] coatings are most often used to protect turbine blades. Gas turbine blades can be coated with thermal spray technology [28].

The hardness of thermally sprayed coatings depends on the chemical composition of the coating and the spraying method. The hardness of the WC-WB-Co coating was found to be between 1200 and 1300 HV_0.1_, and the hardness of the WC-FeCrAl coating was found to be between 1000 and 1100 HV_0.1_ [29]. Microhardness values from HV = 650 to 1021 were obtained for WC-12Co (88wt%WC–12wt%Co) coatings containing phases WC, W_2_C and Co sprayed using the HVOF method [30]. In the works of Myalska et al. [31] and Gaceb et al. [32], the hardness of the thermally sprayed NiCrBSiFeC coating was HV_0.1_ = 753 ± 115. In turn, research by Rachidi et al. [33] showed that the hardness of the NiCrBSi coating by the thermal spraying technique using SuperJetEutalloy reached an average of 800HV_0.3_, and the NiCrBSi—60% WC coating reached the hardness of 1200HV_0.3_. In the work [34], Cr_3_C_2_-25NiCr and WC-10Co-4C coatings were tested. The hardness of the first coating fluctuating between 900–1050 HV_2.94N_ and the second between 1000–1200 HV_2.94N_. The hardness of the WC-CoCr coating produced using the high-velocity oxygen–fuel spraying (HVOF) method was above 1000 HV_0.3_. The variation in microhardness was only 215.7 HV_0.3_ [35]. The hardness of the WC-4Co-10Cr coating sprayed using the HVOF method reached values from 1177 to 1256 HV_0.3_ [36]. The WC-12Co coating thermally sprayed onto the TC18 substrate achieved the hardness of 1018–1213 HV_0.3_ [37]. Tungsten carbide has a very high hardness of approximately 2200–2800 HV, while the hardness of HVOF coatings made of WC-Co-based powders is reported in research works as significantly lower compared to the hardness of tungsten carbide, from 866 to 1226 HV [38]. The reduction in hardness is influenced by the mechanism of the thermal spray process, in which very high temperatures lead to decarbonization of WC carbide. The presence of the bonding phase, i.e., Co, Ni, Cr, which has a lower hardness than tungsten carbide, also reduces the hardness level of the sprayed coating. Hardness is also reduced by various artifacts occurring in the structure of the coatings. The presented data indicate that the microhardness range obtained for coatings based on tungsten carbide and chromium carbide ranges from 600 to 1300 HV01. The level of microhardness depends not only on the chemical composition of the coating but also on the method of coating deposition.

Research shows that hardness is related to erosion resistance. The erosion rates were found to increase with the increase in the alloy bulk hardness [39]. The wear resistance of the hard metal coatings is highly dependent on its composition, carbide grain size, porosity, toughness and hardness value [40]. In turn, the results of the work of Cesanek and Schubert show that the abrasion resistance is not directly proportional to hardness [41]. The effect of hardness on the abrasion resistance of particles was studied in the work of Sundararajan [42]. It was found that abrasion resistance has a different relationship with hardness. The impact of hardness on abrasion resistance and the impact of such parameters as impact velocity, impact angle and the size and shape of the particles were studied in the work of Oka and Hutchings [43]. It was found that hardness is an important parameter determining abrasive wear resistance. Sheldon also points out that Vickers hardness is probably one of the most important parameters in determining erosion resistance [44]. Studies on the influence of hardness, thickness of the protective coating and the coating structure on the erosion resistance of turbine blades are presented in the work by Wang et al. [45]. It was found that there is a critical coating thickness of at least 0.02 mm that is necessary to protect the surface. Cracks and discontinuities in the coating reduce erosion resistance.

The key research issue is the possibility of assessing the relationship between the influence of hardness and the erosion resistance of the coating. The presented data indicate that there is no clear relationship between erosion resistance and the level of microhardness. Most studies indicated an increase in erosion resistance with an increase in coating microhardness. However, it was found that, as the size of the eroding particle increases and the speed of the abrasive stream increases, the nature of the wear of the cermet changes to a more plastic one. This means that there is a research gap regarding the impact of various environmental parameters on the coating’s resistance to erosion in dynamic operating conditions.

In the work by Levy and Wang, it was determined that a small grain size, low porosity and the absence of cracks were the microstructural features that enhanced erosion resistance [46]. Hardness levels and the composition and distribution of the second phase, i.e., hard particles, had less effect on coating performance. In turn, the appropriate level of hardness of the HVOF coating ensures erosion resistance [47]. In the work by Wen, the resistance of steel to erosion by sand blasting [48] was examined. The results show that the increased hardness resulted in lower erosion rates because the increase in hardness provides resistance to penetration and results in a lower erosion rate. Thermally sprayed coatings consist of hard ceramic particles with a tough metallic binder. The ceramic particles provide excellent wear and corrosion resistance due to a high hardness and chemical stability. The metal matrix is usually ductile and provides good wetting and adhesion characteristics that, in return, give fracture toughness to the coating. This structure of coatings, especially those with almost nanometric grains, provides a very good resistance to abrasion and corrosion [49].

The great usefulness of thermal spray methods for anti-erosion and anti-corrosion surface protection was confirmed in the review work by Mehta et al. [50]. A study of the effect of particle impact velocity and the hardness of the material on the wear rate was presented in the work of Kosa and Göksenli [51]. Erosion resistance increased with increasing hardness. In turn, the study presented in the work [52] shows that an increase in plasticity leads to a greater resistance to the erosive impact of particles. This is the result of absorbing the kinetic energy of impacting particles, causing plastic deformation at the surface while maintaining the material within the fracture strain limits. It was observed that, under certain conditions, the erosion rate of WC/Co cermets decreases monotonically with the increase in the volume fraction of reinforcement [53]. However, it was not clear-cut because, with the increasing volume fraction of WC for a WC-Co cermet following an impact by a slurry of 100 μm silicon carbide particles at 40 ms^−1^ and at impact angles of 90°, the erosion increased to a certain WC content and then decreased.

The presented work focuses on issues related to the protection of the surfaces of technical fan blades exposed to the erosive impact of the industrial environment. There is a variable relationship between erosion resistance and hardness. Despite this, it should be noted that, in general, coatings are resistant to erosion precisely due to their high hardness. Therefore, the hardness parameter should be considered the most important in assessing the suitability of a coating for erosion protection. However, within the range of hardness obtained for cermetal coatings reported in the literature, research is needed to select individual types of coatings for specific operational purposes.

The aim of this research was to produce a thermally sprayed coating that would effectively protect the material of the rotor blades against damage caused by particles contained in the air exhausted by industrial fans. The rationale for undertaking this research was the possibility of reducing the weight of fan blades and, consequently, reducing energy consumption. Thermally sprayed coatings, due to their small thickness compared to, e.g., hard facing coatings, do not excessively load the blades and, therefore, consume less energy. At the same time, an appropriately selected chemical composition of the coating, ensuring a high hardness, should effectively protect the rotor blade material against damage. One of the most commonly used coatings for an anti-erosion surface protection are carbide coatings [54,55,56,57].

This work examined WC-Co and Cr_3_C_2_ coatings in a Co, Ni, Cr matrix and their various compositions, determining their hardness and structure. The high hardness was the basis for selecting the coatings that best met the desired feature of the resistance to particle impact in the medium centrifuged by fans. The use of diverse, new chemical compositions of coatings based on tungsten carbide and chromium carbide and a mixture of these carbides enabled the verification of the hypothesis, determining the possibility of increasing resistance to erosive wear through the synergistic effect of the carbide composition.

## 2. Materials and Methods

This study examined thermally sprayed coatings on a substrate made of A03590 casting aluminum alloy with the chemical composition shown in Table 1.

Carbide coatings based on the Cr_3_C_2_-NiCr powder and WC-Co powder were selected for testing using various application parameters. Coatings made of NiCrSiB composite materials with the addition of E = 2.5%Fe + 3.1%Si + 0.4%C + 2.1%Bi + Ni residue were also deposited. Coatings were also made from the following compositions: NiCrSiB + 5%Fe—nanoparticles, NiCrSiB + 5%Cr—nanoparticles, NiCrSiB + 2.5%Fe + 2.5%Cr—nanoparticles. In addition, a coating was produced from a mixture of Cr_3_C_2_ and WC-Co and an Inconel 625 (Table 2 and Table 3). The differences in the chemical composition of the coatings were reflected in their different levels of microhardness. The microhardness value was adopted as a parameter for selecting the optimal coating, resistant to the erosive effects of the environment. The basic criterion for the suitability of the coatings was a high microhardness value. Microhardness was tested using the Haneman method on the cross-section of samples after grinding and polishing. Microhardness was measured on polished samples under a load of 200 G using a Microhardness measuring microscope (PMT-3)–Vickers microhardness tester (from Russian manufacturer). In addition to microhardness tests, the structure of the coatings was observed. Samples for structure observation were cut perpendicularly to the thermally sprayed surface using a Leco ball with a diamond disc cooled with oil diluted with water. The cut sections were sanded on 220–4000 grit sandpaper. Then, the samples were polished using an Al_2_O_3_ OPS suspension and diamond pastes with a gradation of 6 µm, 3 µm and 1 µm, manufactured by Struers (Kraków, Poland). Samples for observation using a scanning electron microscope (SEM) were embedded in a special conductive material before preparation for testing. The structure of the samples was examined using a STEREOSCAN 420 scanning electron microscope(Manufacturer CAMBRIDGE, UK). The chemical composition of the samples was examined using the EDX method with a scanning electron microscope. In the first stage of the research, the microhardness of the coatings was measured. Then, 7 coatings with the highest level of microhardness were selected and tested for resistance to the erosive impact of the environment with corundum particles under the operating conditions of an industrial fan rotor. The coatings were sprayed onto individual rotor blades. Each coating was deposited on one of the rotor blades. The test rotor operated at a speed of 3000 rotations per minute, and it was exposed to the veneer paper of panels that contained corundum. The operating time of the rotor in operational conditions was 2.5 months. After this period of operation, the rotor was dismantled, and the coatings were examined for wear and tear and assessed for pitting, cracks and damage. After assessing the tested rotor, the coating that showed the least damage was selected.

## 3. Results

**Figure 1:**
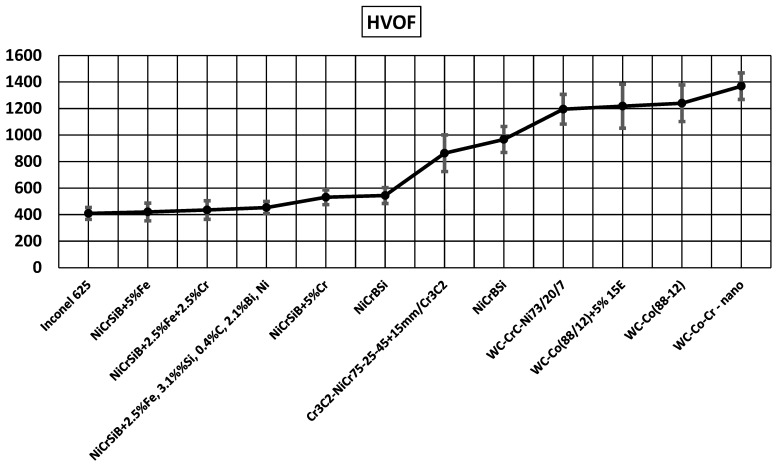
Microhardness of coatings produced by the HVOF method.

**Figure 2:**
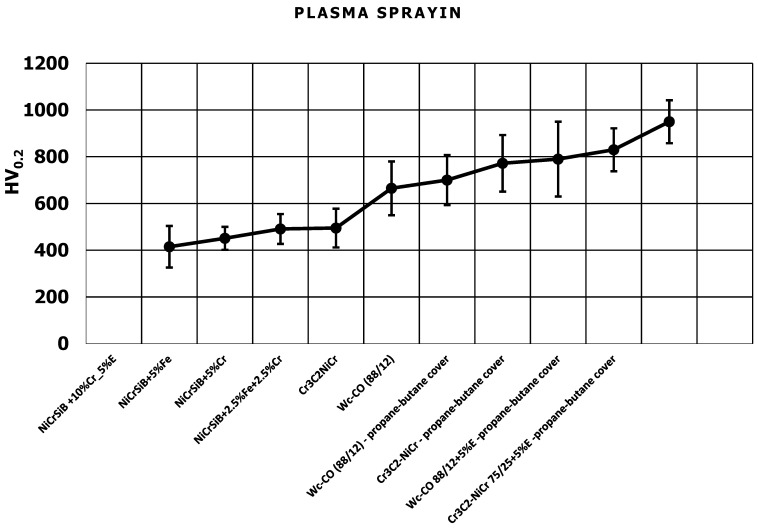
Microhardness of plasma spray coatings, E = 2.5%Fe + 3.1%Si + 0.4%C + 2.1%Bi + Ni rest.

Figure 1 and Figure 2 show the results of microhardness measurements of coatings applied to the AK9 substrate. The microhardness of coatings sprayed using the HVOF method is shown in Figure 1, and the microhardness of plasma sprayed coatings is shown in Figure 2. The microhardness of coatings sprayed using the HVOF method showed higher values than the microhardness of plasma spray coatings. The highest microhardness, among the coatings produced by the HVOF method, was achieved by the WC-Co-Ni coating sprayed from nanometric powders. In the case of plasma sprayed coatings, the highest hardness was demonstrated by the Cr_3_C_2_-NiCr +5%E composite coating produced in a propane–butane cover.

Seven coatings with the highest microhardness were selected for further testing. The chemical composition of the selected coatings and the type of deposition process are presented in Table 4.

**Figure 3:**
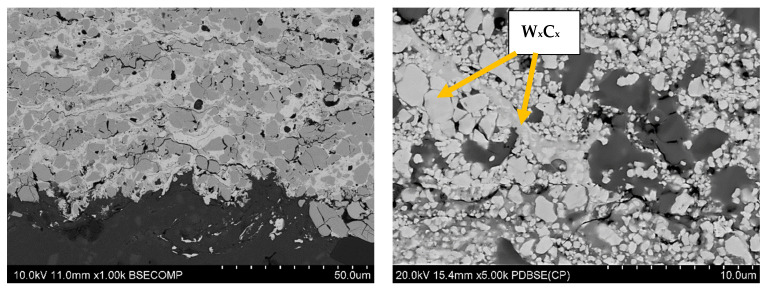
Coating No. 1: WC-Co-Cr, HVOF (HV_0.2_ = 1369) (W_x_C_x_—possible W_2_C, WC).

**Figure 4:**
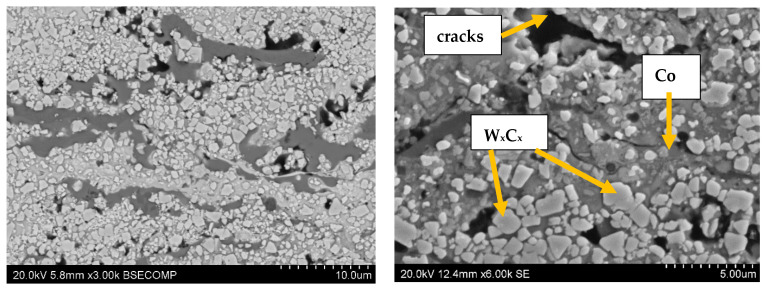
Coating No. 2: WC-Co, HVOF (HV_0.2_ = 1240) (W_x_C_x_—possible W_2_C, WC).

**Figure 5:**
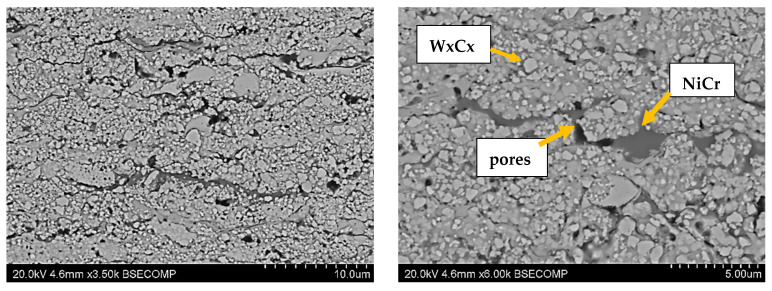
Coating No. 3: WC-Co + 5% NiCrSiB, plasma spray in a propane–butane shield (HV_0.2_ = 1218), equiaxed W_x_C_x_ carbides (possible W_2_C, WC), NiCrSiB matrix.

**Figure 6:**
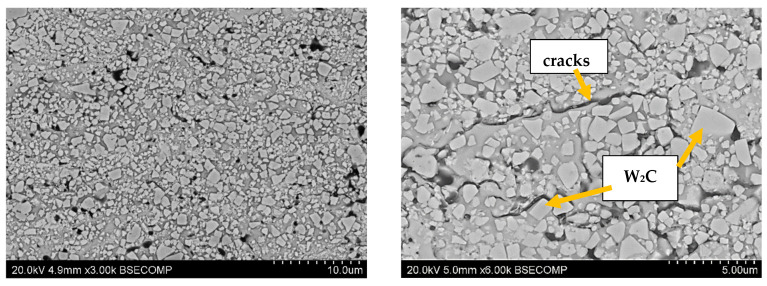
Coating No. 4: WC-Co-CrC-Ni, HVOF (HV_0.2_ = 1195).

**Figure 7:**
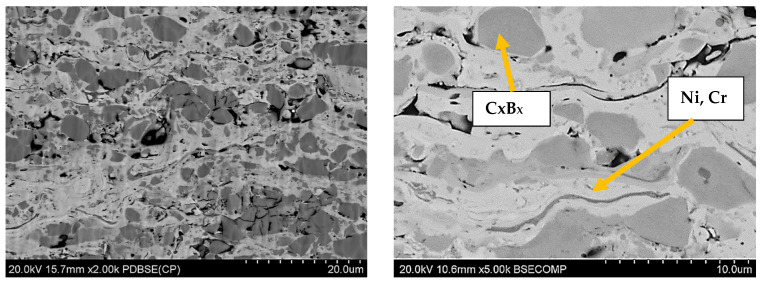
Coating No. 5: NiCrBSi, HVOF (HV_0.2_ = 967) (Cr_x_B_x_—possible CrB, Cr_3_B_2_, Cr_3_B).

**Figure 8:**
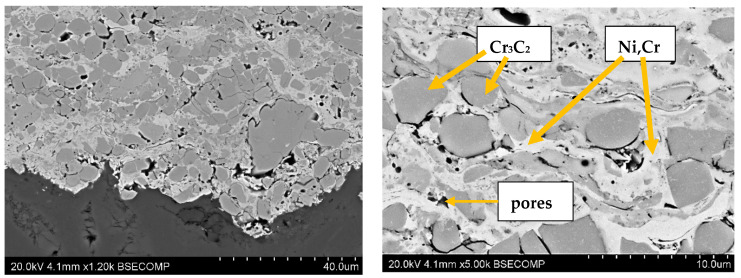
Coating No. 6: Cr_3_C_2_-NiCr + 5%NiCrBSi, plasma-spray from nanopowders in a propane–butane shield (HV_0.2_ = 950).

**Figure 9:**
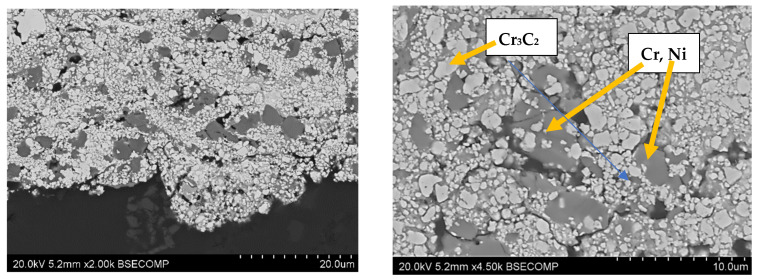
Coating No. 7: Cr_3_C_2_—NiCr, HVOF (HV_0.2_ = 863).

The structures of the coatings with the highest microhardness are shown in Figure 3, Figure 4, Figure 5, Figure 6, Figure 7, Figure 8 and Figure 9.

**Figure 10:**
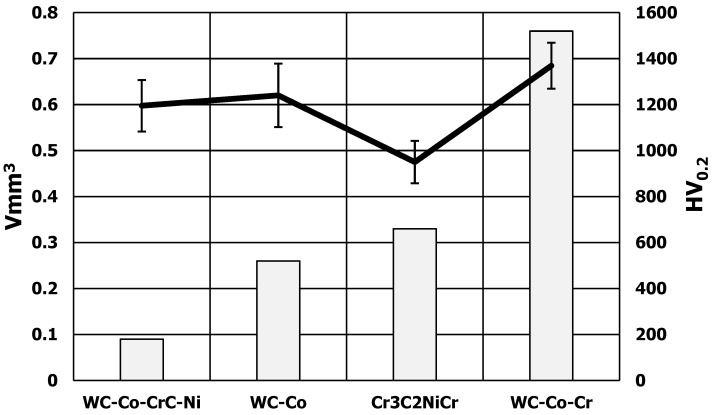
Comparison of the volume of erosion craters of thermally sprayed coatings against the microhardness results of the coatings.

Erosive wear resistance tests were carried out using a sanding device operating at an air pressure in the range of 1 ÷ 6 bar. The volume of material removed during the test was taken as a measure of the erosion resistance of the tested coatings (Figure 10).

The abrasion results of the Cr_3_C_2_NiCr coating did not correlate with the results of the tungsten carbide-based coatings. A correlation was obtained between the hardness of coatings based on WC and abrasion. The higher the hardness of the coating, the larger the craters and the volume of material removed. It seems that a higher plasticity of the coating ensures a lower degree of carbide knockout by the particles of the centrifuged medium. The WC-Co-CrC-Ni coating, sprayed using the HVOF method (HV_0.2_ = 1195), showed the greatest resistance to the erosive impact of particles. The resistance of the coatings to the particle impact was on average 18 times greater than the resistance of the AK9 alloy substrate.

**Figure 11:**
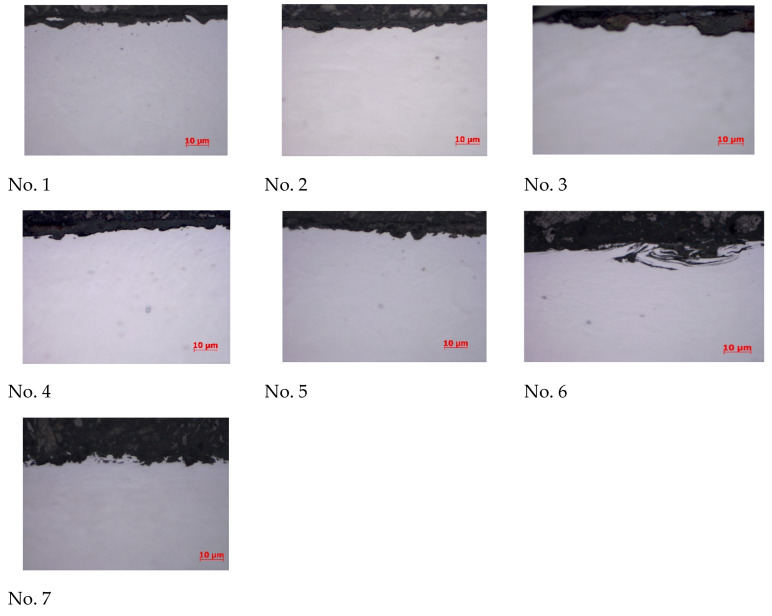
Surface profiles of coatings that were applied to the blades of a rotor tested in industrial conditions.

The coatings with the highest hardness were applied to the blades of the test rotor, which operated in industrial conditions and were exposed to corundum particles. In Figure 11, the surface profiles of the coatings applied to the rotor blades tested in industrial conditions are presented. All profiles show pitting craters caused by the impact of the hard particles of the centrifuged medium.

A macroscopic inspection of the rotor blades was also carried out after the industrial test. Tests of the rotor blades after operation in industrial conditions showed that the least wear was observed for the following coatings: No. 2—WC-Co (HVOF), HV_0.2_ = 1240; No. 4—WC-Co-CrC-Ni (HVOF), HV_0.2_ = 1195; and No. 7—Cr_3_C_2_-NiCr (HVOF), HV_0.2_ = 863.

**Figure 12:**
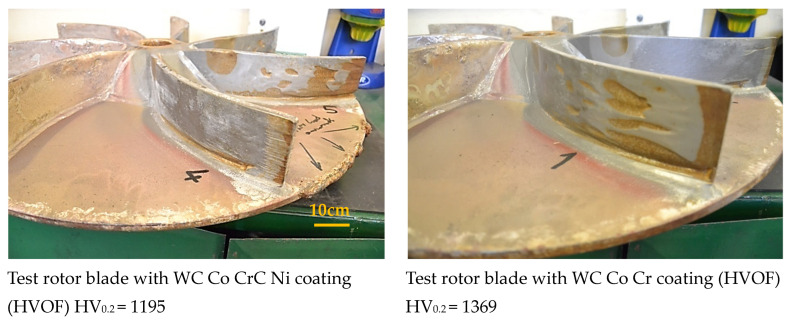
Macroscopic view of the rotor blades after an industrial test.

Among the mentioned coatings, the surface that was least damaged and destroyed by the centrifuged medium was coating No. 4, made of tungsten carbide and also containing chromium carbide WC-Co-CrC-Ni, deposited using the HVOF method, with a microhardness value of HV_0.2_ = 1195 (Figure 12).

The microhardness of coating No. 4 is 12% lower than the value of the hardest WC-Co-Cr coating deposited from nanopowders using the HVOF method (HV_0.2_ = 1369) (Table 4). Nevertheless, it shows a greater resistance to impact erosion by hard corundum particles.

## 4. Discussion

Tungsten is an excellent conductor of electricity and heat, and it becomes a superconductor at low temperatures. It is the element with the highest melting point of all metals. This value is 2870 °C. The density of tungsten carbide is 15.5 g/cm^3^. This structure significantly increases the strength of the material and improves its thermal conductivity. Another important parameter of tungsten carbide is its tensile and compressive strength, which is directly responsible for the strength of the material during its use. The Young’s modulus in this case is 530–700 GPa. This means that tungsten carbide subjected to stresses of the indicated value will not undergo permanent deformation.

Chromium carbides are also characterized by a high resistance to erosive and corrosive wear [58]. The hardness of chromium carbide coatings is in the range of 770–1600 HV, depending on the production method [59,60]. The Cr_3_C_2_ compound has exhibited the highest hardness value and the maximum Cr-C bond strength. The influence of Cr-C bond strength and the density of Cr_3_C_2_ play a remarkable role. The Cr_3_C_2_ exhibited the highest strength under the applied pressure. It has also exhibited the best mechanical properties compared to Cr_7_C_3_ and Cr_23_C_6_ [61]. The presence of the metal binder (Co, Ni and Cr) and its different content in the coatings affects the hardness of the coatings.

In the tests carried out on the susceptibility to the impact erosion of particles, the highest hardness was obtained by coating No. 1—WC-Co-Cr, HVOF (HV_0.2_ = 1369), and the lowest was obtained by coating No. 7—Cr_3_C_2_—NiCr, HVOF (HV_0.2_ = 863). It was also found that coating No. 4—WC-Co-CrC-Ni, HVOF (HV_0.2_ = 1195)—has the best wear resistance.

This confirms the results of the study [62], which showed that the WC-based coating has a better resistance to abrasive wear compared to the CrC-based coating. In addition, the carbide-based coatings applied via the HVOF method have an excellent wear resistance compared to other application methods. The results are also confirmed by the research work [41], which suggests that there is no simple relationship between the resistance to erosive wear and the hardness level. Overall, the high hardness provides wear resistance. However, depending on the method of impact of erosive particles, the speed of their impact on the substrate, the impact angle, the particle size and other parameters, a complex nature of the relationship between abrasive wear resistance and the material and strength parameters of the coating can be expected.

The good thermal conductivity of tungsten causes a very rapid reduction in the particle temperature during the production of coatings. This may contribute to an increase in the fraction of unmelted particles. The carbide composition may also be transformed as a result of decarbonization. In this kind of cermet coating, some brittle phases unavoidably appear (e.g., W_2_C, W and Co_x_W_y_C_z_) due to the decomposition and decarburization of WC in the high-temperature thermal spraying flame [63]. The melting of carbide particles and their plastic deformation in thermal spraying processes, caused by the strong impact of particles on the substrate, affect the hardness of the coating. The particles decarbonize and also break down [64]. As a result of decarbonization, the following phases may appear in the structure: WC, W_2_C, W, Co_3_W_3_C or Co [65]. In the case of chromium carbides, depending on the C/N ratio, the following phases are formed: Cr_3_C_2_, Cr_7_C_3_, CrO_2_, Cr _6.2_C_3.5_ or Cr_3_C_1.5_ [66]. Changes in the phase composition affect the hardness level of the coatings and their resistance to abrasive wear resulting from interaction with particles of the centrifuged medium.

It is assumed that the reason for the almost two-fold acceleration of the wear process of the WC-17%Co coating compared to WC-12%Co may be the reduction in the WC carbide share at the expense of W_2_C and (W,Co)_6_ C. The thermal dissolution of WC is primarily responsible for the occurrence of W_2_C. The semicarbide (W_2_C) crystals are observed to grow epitaxially on the affected WC grain and extend radially with progressively thinning cross-sections [67].

The results of the erosive wear tests conducted allow for us to conclude that the chemical composition of multi-component coatings based on WC-Co has a significant impact on their resistance to erosive wear. The synergistic interaction of tungsten carbide and chromium carbide results in the best resistance to abrasive wear by centrifuged particles (Figure 12). It reduces the wear parameter almost ten times compared to other chemical compositions of coatings. Abrasion tests indicate that, for tungsten carbide-based coatings, sand impact craters increase with increasing coating hardness. This is probably related to a greater susceptibility to cracking of hard WC grains and a lower plasticity of the coating [68]. The micro-cracks at grain boundaries significantly influence both the sliding and abrasive wear rate of WC-Co coatings. This paper hypothesized that the crack propagation for smaller carbides proceeds through the interlamellar (i.e., intergranular) boundary, whereas for larger carbides, cracks propagate through the carbide (i.e., transgranular). Yuan et al. [69] concluded that submicron-sized WC particles uniformly distributed at the interfaces could cause deflection or impede fracture. This indicates that the more homogeneous the coating structure, the more resistant it should be to erosive influences.

The work [70] concluded that the average friction coefficient depends strongly on the cermet fraction, exhibiting a maximum value of 0.64 at 50% WC-Co/Cu content. The wear coefficient values decrease almost linearly with increasing cermet fraction.

Tests of cermet coatings based on WC and Cr_3_C_2_ do not reveal simple relationships between wear resistance and the hardness and structure of the coating. The coatings contain numerous defects in the form of discontinuities, unmelted grains and other artifacts that affect the hardness of the coating. The size of powders and coating production conditions also affect their properties [71,72,73].

The tests carried out showed that coatings based on WC powder deposited using the PS method showed lower hardness than coatings deposited using the HVOF method. The WC-Co coating deposited using the HVOF method had a hardness of HV_0.2_ = 1240, and the one deposited using the PS method had a hardness of HV_0.2_ = 830. Coatings based on NiCrSiB powder with various admixtures had a comparable hardness level both in the case of deposition using the HVOF method and in the case of deposition using the PS method. It was a level in the range of HV_0.2_ = 400–500. Coatings based on Cr_3_C_2_ powder showed a hardness of HV_0.2_ = 950 when deposited using the PS method, and HV_0.2_ = 863 when deposited using the HVOF method. In the industrial test, four coatings were selected that were most resistant to the erosive impact of dynamic particle streams. These were coatings deposited using the HVOF method, No. 2—WC-Co (HV_0.2_ = 1240), No. 4—WC-Co-CrC-Ni (HV_0.2_ = 1195) and No. 7—Cr_3_C_2_-NiCr (HV_0.2_ = 863), and a coating deposited using the PS Cr_3_C_2_NiCr method (HV_0.2_ = 950). Among the four coatings most resistant to erosion in industrial conditions, one coating was selected that also showed the highest resistance in the sandblasting test. It was coating No. 4—WC-Co-CrC-Ni.

In this case, coating No. 4 also turned out to be the most resistant to damage of centrifuged corundum particles (No. 4—WC-Co-CrC-Ni—sprayed using the HVOF method (HV_0,2_ = 1195)). It was not the coating with the highest hardness, but it belonged to the group of the hardest WC-based carbide coatings sprayed onto the surface of the rotor blades. The qualitative assessment of the damage to the blades caused by the corundum particles as well as the similar level of hardness of the tested coatings and the discontinuities present in the coatings are reasons to consider this coating as potentially offering the best anti-erosion properties. The performed technical test indicates the suitability of coating No. 4 for operation in difficult conditions of exposure to impacts of hard corundum particles found in the centrifuged environment.

Coating No. 4—WC-Co-CrC-Ni—had a composition similar to the WC-Co-Cr coating tested in the work by Jonda and Łatka [74]. The hardness of both coatings was practically the same. The hardness of the WC-Co-CrC-Ni coating was HV_0,2_ = 1195, and the hardness of the coating tested in the work of Jonda and Łatka, WC-Co-Cr, was HV = 1198. In the work of Jonda and Łatka, it was shown that WC-Co-Cr is the most promising candidate for the further dry sliding, erosion and cavitation resistance coating. It was also characterized by a considerable hardness, a relatively good fracture toughness and a high value of elastic modulus.

The convergence of the results confirms the data obtained and indicates that the chemical composition of coating No. 4 is favorable for practical applications. Studies of carbide coatings produced by the HVOF method also showed that, in the case of the WC-CrC-Ni coating, the hardening of the matrix caused by the dissolution of Cr is accompanied by an increase in the matrix’s plasticity, which prevents carbides from detaching from the matrix [75]. According to Korobov et al. [76], the fine structure of the WC-CrC-Ni coating increases the specific surface area of carbide particles and, consequently, the required crack propagation energy. This improves their resistance to chipping and, consequently, to cavitation compared, for example, to the coarser WC-CoCr coating. Tests on the properties of WC−Co, WC−Co−Cr_3_C_2_ and WC−Co−TaC showed that the addition of Cr_3_C_2_ or TaC improves the hardness and fracture toughness of the obtained sintered compacts [76].

The increasing use of surface protection is associated with reduced operating costs of protected devices [77]. In particular, thermal spraying methods, which have multiple varieties and enable the spraying of protective coatings in various conditions, present a favorable level of costs and high effectiveness of protective layers. The hardness achieved in the case of carbide coatings based on WC and Cr_3_C_2_ is approximately 1000–1200HV, and in many cases, it is sufficient to protect the surface against particle impact erosion.

## 5. Conclusions

Based on the research conducted, the following final conclusions were made.

1. It was found that the coatings most resistant to particle impact erosion were WC-Co, HV_0.2_ = 1240; WC-Co-CrC-Ni, HV_0.2_ = 1195; and Cr_3_C_2_-NiCr, HV_0.2_ = 863 deposited by the HVOF method and coating Cr_3_C_2_NiCr deposited by the PS method.
2. The WC-Co-CrC-Ni coating showed the highest, simultaneous resistance to the impact of a stream of corundum particles and SiO_2_ particles.
3. The greatest resistance of the WC-Co-CrC-Ni coating to the erosive impact of dynamic particle streams results from the synergistic effect of tungsten and chromium carbides.

## Figures and Tables

**Table 1:** Chemical composition of A03590 alloy.

Si	Cu	Mg	Mn	Ti	Zn	Fe	Al
8.5–10.5	0.3	0.25–0.4	0.25–0.5	0.15	0.2	0.6	rest

**Table 2:** Chemical composition of powders and parameters of thermal spray coating.

Powder Composition	Plasma Spray Parameters				Coating Thickness [µm]
	Ar [L/h]	Voltage [V]	Current [A]	Distance [mm]	
WC-Co 88/12	3000	52	500	90	300 ± 20
WC-Co 88/12 (+propane–butane cover)	3000	52	500	90	
NiCrSiB	2500	48	480	90	
NiCrSiB + 5% Fe	2500	48	480	90	
NiCrSiB + 5% Cr	2500	48	480	90	
NiCrSiB + 2.5% Fe + 2.5% Cr	2500	48	480	90	
Cr_3_C_2_—NiCr 75–25	3000	52	500	90	
Cr_3_C_2_—NiCr 75–25 -45+ (+propane–butane cover)	3000	52	500	90	
WC-Co + 5%(NiCrFeBSi) (+propane–butane cover)	3000	52	500	90	

**Table 3:** Chemical composition and parameters of HVOF deposition.

Powder Composition	HVOF Spray Parameters					Coating Thickness [µm]
	O_2_ [L/min]	Kerosene [L/h]	N_2_ [L/min]	Distance [mm]	Powder [g/min]	
WC-Co 88-12	944	25.5	9.5	370	92	300^+80^
NiCrSiB	920	21.5	11	355	95	
NiCrSiB+5%Fe—nanopowder	920	21.5	11	355	95	
NiCrSiB+5%Cr—nanopowder	920	21.5	11	355	95	
NiCrSiB + 2.5%Fe + 2.5%Cr—nanopowder	920	21.5	11	355	95	
Cr_3_C_2_-NiCr 75–25	850	24	9.5	370	65	
WC-Co + 5%(NiCrFeBSi)	944	25.5	9.5	370	92	
WC-CrC-Ni 73/20/7	944	24	10	370	75	
WC-Co-Cr 86/10/4 nanopowder	944	25.5	9.5	370	92	300^+50^
Ni-Cr-B-Si	944	24	10	370	75	300^+50^
Inconel 625	944	24	10	370	75	300^+50^

**Table 4:** Chemical composition of coatings deposited on rotor blades intended for industrial tests.

Rotor Blade No	Chemical Composition	Thermal Spray Method	HV_0.2_
1	WC-Co-Cr	HVOF	1369 (±100)
2	WC-Co	HVOF	1240 (±138)
3	WC-Co + 5% NiCrBSi	plasma spray	1218 (±166)
4	WC-Co-CrC-Ni	HVOF	1195 (±112)
5	Cr_3_C_2_-NiCr + 5% NiCrBSi	plasma spray	967 (±98)
6	NiCrBSi	HVOF	950 (±92)
7	Cr_3_C_2_-NiCr	HVOF	863 ± (138)

## Data Availability

The original contributions presented in the study are included in the article, further inquiries can be directed to the corresponding author.
